# In Vivo Genome-Wide PGR Binding in Pregnant Human Myometrium Identifies Potential Regulators of Labor

**DOI:** 10.1007/s43032-022-01002-0

**Published:** 2022-06-22

**Authors:** Ariel J. Dotts, Derek Reiman, Ping Yin, Stacy Kujawa, William A. Grobman, Yang Dai, Serdar E. Bulun

**Affiliations:** 1 Department of Obstetrics & Gynecology, Feinberg School of Medicine, Northwestern University, Chicago, IL 60611 USA; 2 Department of Bioengineering, University of Illinois at Chicago, Chicago, IL 60612 USA

**Keywords:** Human myometrial tissues, Transcriptome, PGR, Labor

## Abstract

The alterations in myometrial biology during labor are not well understood. The myometrium is the contractile portion of the uterus and contributes to labor, a process that may be regulated by the steroid hormone progesterone. Thus, human myometrial tissues from term pregnant in-active-labor (TIL) and term pregnant not-in-labor (TNIL) subjects were used for genome-wide analyses to elucidate potential future preventive or therapeutic targets involved in the regulation of labor. Using myometrial tissues directly subjected to RNA sequencing (RNA-seq), progesterone receptor (PGR) chromatin immunoprecipitation sequencing (ChIP-seq), and histone modification ChIP-seq, we profiled genome-wide changes associated with gene expression in myometrial smooth muscle tissue in vivo. In TIL myometrium, PGR predominantly occupied promoter regions, including the classical progesterone response element, whereas it bound mainly to intergenic regions in TNIL myometrial tissue. Differential binding analysis uncovered over 1700 differential PGR-bound sites between TIL and TNIL, with 1361 sites gained and 428 lost in labor. Functional analysis identified multiple pathways involved in cAMP-mediated signaling enriched in labor. A three-way integration of the data for ChIP-seq, RNA-seq, and active histone marks uncovered the following genes associated with PGR binding, transcriptional activation, and altered mRNA levels: ATP11A, CBX7, and TNS1. In vitro studies showed that ATP11A, CBX7, and TNS1 are progesterone responsive. We speculate that these genes may contribute to the contractile phenotype of the myometrium during various stages of labor. In conclusion, we provide novel labor-associated genome-wide events and PGR-target genes that can serve as targets for future mechanistic studies.

**Supplementary Information:**

The online version contains supplementary material available at 10.1007/s43032-022-01002-0.

## Introduction 

Preterm birth is a leading cause of infant deaths worldwide [58]. An estimated 15 million babies are born preterm annually across the world [57], with approximately 1 million worldwide deaths from preterm birth (PTB) complications [28]. Racial disparities are present in PTB rates, with African American women exhibiting the highest PTB rates compared to non-Hispanic white women, non-Hispanic Asian women, and Hispanic women over the past 5 years [28]. Despite efforts to prevent PTB, the rate of PTB in the USA continues to steadily increase as recently from 2015 to 2019 [19]. The pathology of PTB is not yet known, and current efforts are only partially successful in prevention. Thus, there is a critical need to understand the pathobiology of PTB to develop preventive or therapeutic strategies for this disease.

The steroid hormone progesterone plays a crucial role in myometrial smooth muscle, such as the establishment and maintenance of pregnancy. Evidence of the importance of progesterone in the maintenance of pregnancy is demonstrated by spontaneous pregnancy loss with progesterone receptor (PGR) antagonists at any stage of pregnancy [2], such as mifepristone (RU486), and the use of PGR modulators, ulipristal acetate, as emergency contraception [16]. Many mammals show a decline in circulating progesterone levels proceeding labor initiation. This decline in serum progesterone led to the hypothesis that the abatement of progesterone initiates labor. However, circulating progesterone levels remain high in humans during gestation, which led to a functional progesterone withdrawal theory whereby progesterone, acting through its receptor, PGR, is no longer functional in maintaining pregnancy through an unknown mechanism [5, 32]. It was proposed that various PGR isoforms played roles in the control of labor [10, 32, 35]. Recent findings support changes in the ratio of PGR isoform B versus A as gestation progresses, with PGR-B dominating in early pregnancy and PGR-A dominating toward the end of pregnancy in human myometrial cells and primate tissues [18, 32, 34, 35]. Not only is this seen in human myometrial cells and primate tissue, but increased myometrial PGR-A is also associated with the progression of gestation and onset of labor in rodents [13, 64].

Furthermore, overexpression of PGR-B in a mouse model was shown to prolong gestation leading to delayed parturition with no comparable changes in serum progesterone levels to controls [39]. In contrast, overexpression of PGR-A resulted in increased fetal loss. These findings suggest that differences in the ratio of PGR isoform B versus A are essential for the switch of quiescent myometrium to contractile myometrium. Despite this knowledge, direct target genes of PGR in pregnant term laboring and non-laboring human myometrium have not been differentiated to determine possible molecular mechanisms of PGR in labor.

Transcriptomic profiles, found by RNA-sequencing (RNA-seq) using a low number of reads, from term pregnant not laboring and laboring myometrial tissues are distinct, and a significant number of differential transcripts have been previously identified [8, 47]. Additionally, distinct transcriptomes of myometrium from pregnant and nonpregnant women are also observed. Integrating distinct and shared PGR binding sites with differentially expressed genes found between the two groups has provided insight into important PGR target pathways in pregnancy and, potentially, the labor cascade [59]. Yet, further information is required to investigate the role of PGR on the transcription of genes necessary for the switch from quiescent myometrium to active myometrium in labor.

In this study, we explored this phenomenon in vivo using myometrial tissues from women who are term in labor (TIL; > 37 weeks with signs of labor) and term not in labor (TNIL; > 37 weeks with no signs of labor) to determine the progesterone/PGR target genes which are differentially expressed between pregnant myometrial tissues. In vivo assessment of genome-wide PGR binding using the chromatin immunoprecipitation (ChIP)-sequencing (seq) procedure using tissue versus cultured cells has been technically challenging but, cultured cells do not accurately model a laboring versus non-laboring phenotype. Thus, we evaluate whether functional PGR binding in myometrial tissue drives labor by association to genes that play a role in myometrial activation and contraction.

## Results

### Clinical Characteristics of Myometrial Specimens

The characteristics of the pregnant patients from whom the myometrial tissues were collected and used for experiments are described in Table 1. The RNA-seq experiments were conducted on 11 samples (TNIL, *n* = 7; TIL, *n* = 4), PGR ChIP-seq on 6 samples (TNIL, *n* = 3; TIL, *n* = 3), and histone ChIP-seq on 5 samples (TNIL, *n* = 3; TIL, *n* = 2). Results showed no statistical differences in maternal age, parity, BMI, and gestational age among the groups. Our pool of patients was racially diverse, consisting of African American, Latina, and Caucasian women.

**Table 1:** Clinical characteristics of myometrial specimens

	Maternal age (years)	Parity	bmi (kg/m^2^)	GA at delivery (weeks)	African-American (Non-Latina)	Caucasian (Non-Latina)	Latina
RNAseq							
TNIL (n = 7)	33.14 ± 1.16	1.14 ± 0.46	31.33 ± 2.05	39.17 ± 0.08	n = 2	n = 5	n = 0
TIL (n = 4)	35.75 ± 2.56	0.75 ± 0.25	31.33 ± 2.76	39.25 ± 0.38	n = 0	n = 3	n = 1
p-value	0.403	0.473	1.000	0.851			
PGR ChlPseq							
TNIL (n = 3)	34.67 ± 2.33	1 ± 0.58	29.01 ± 3.45	38.53 ± 0.68	n = 0	n = 2	n = 1
TIL (n = 3)	37.00 ± 4.16	0.670.33	33.02 ± 2.55	38.70 ± 0.40	n = 0	n = 1	n = 2
p-value	0.657	0.649	0.407	0.845			
Histone ChlPseq							
TNIL (n = 3)	33.33 ± 2.33	2.33 ± 0.33	31.76 ± 4.50	39.00 ± 0.00	n = 1	n = 2	n = 0
TIL (n = 2)	39.00 ± 4.00	1.00 ± 0.00	35.682.40	39.40 ± 0.10	n = 0	n = 1	n = 1

Data are expressed as mean ± SEM. *TNIL*, term not labor; *TL*, term in labor; *BMI*, body mass index; *GA*, gestational age *p*-value are from *t*-tests between TNIL and TIL for each experiment

### Distinct Transcriptomes of TIL and TNIL Myometrial Tissues

**Fig. 1:**
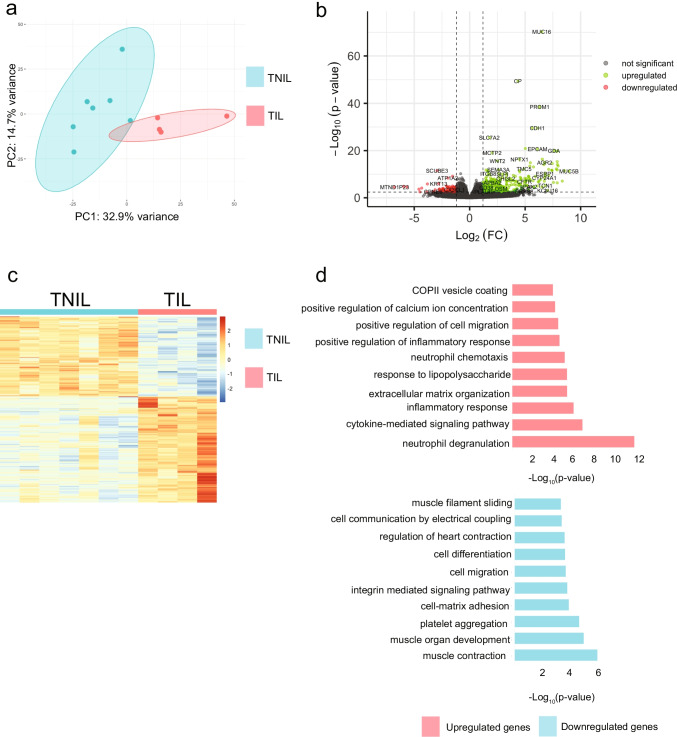
Distinct transcriptomes of TIL and TNIL myometrial tissues. **a** PCA of TNIL (blue) and TIL (pink) samples show distinct clustering. **b** Volcano plot showing all detected transcripts with significant genes (FDR < 0.05) shown as upregulated (green) or downregulated (red) in TIL generated by DESeq2 analysis. **c** Heatmap of significant differentially expressed genes in TNIL (blue) and TIL (pink). **d** Gene ontology of genes upregulated (pink) and downregulated (blue) in labor

To broaden our understanding of the dynamics between TIL and TNIL myometrial tissues, samples were subjected to RNA-seq for genome-wide transcriptomic profiling. Overall transcriptomic profiles show distinct clustering by labor status as demonstrated by principal component analysis (PCA) (Fig. 1a), suggesting significant transcriptomal differences between TIL and TNIL myometrial tissues. Using a cutoff of a false discovery rate < 0.05, 1414 genes were found to be significantly differentially expressed (DEGs). Expression of 605 were found to be higher in TNIL and 809 genes higher in TIL tissues, coined downregulated and upregulated in labor, respectively (Fig. 1c). Among all the DEGs, the most upregulated gene in labor was Mucin 5B (MUC5B), a gel-forming mucin found in cervical mucus [17] (Fig. 1b). The second most upregulated gene was progestagen-associated endometrial protein (PAEP), a secreted glycoprotein produced by the endometrium during pregnancy. Other top upregulated genes were found to serve various biological processes, such as ion transport, innate immunity, and ligand binding (Supplemental Table 1). The most downregulated gene in labor was acrosomal protein (KIAA1210), followed by CUB and Sushi Multiple Domains 1 (CSMD1). Among the most downregulated genes, calcium-binding genes were highly prevalent along with G protein-coupled receptor activity genes (Supplemental Table 2).

**Table 2:** Enriched motifs in TIL and TNIL myometrium H3K27ac peaks from distal regions

Group	Histone Mark	Number of Enriched Motifs	List of Uniquely Enriched Motifs
TIL	H3K27ac	168	AP2-gamma, Bcl11a, bHLHE40, BORIS, CLOCK, EFIF, ERG, FOXK1, Gfi1b, HLF, Hoxc9, MyoD, NFAT, p63, Rfx6, RUNX-AML, RUNX, STAT4, STAT6, Tcf21, TEAD3, TR4
TNIL	H3K27ac	161	E2F, EBNA1, HIF-la, HIF2a, MITF, Nr5a2, p53, PAXS, SF1, Six4, Sp1, Srebp1a, Srebp2, ZFX

Gene ontology analysis of the significant DEGs indicated that genes upregulated in labor are enriched in inflammation and cytokine signaling pathways, consistent with labor associating with inflammatory response in myometrium [4] (Fig. 1d). In contrast, genes downregulated in labor are enriched in terms related to muscle contraction, actin-filament based movement, cell matrix adhesion, and integrin signaling (Fig. 1d). The muscle contraction pathway consisted of protein-encoding genes for tropomyosins, myosin light and heavy chains, calcium voltage-gated channel subunits, integrins, and actins. All significant contributors to smooth muscle contraction [40, 43, 46]. These findings suggest that the muscle contraction and actin movement enriched pathways found from the DEGs increased in TNIL may be preparing the uterus for myometrial contractions required for labor, whereas these processes have already occurred in the TIL group.

### Differential Genome-Wide PGR Binding Between Term in Labor and Not in Labor Myometrium

**Fig. 2:**
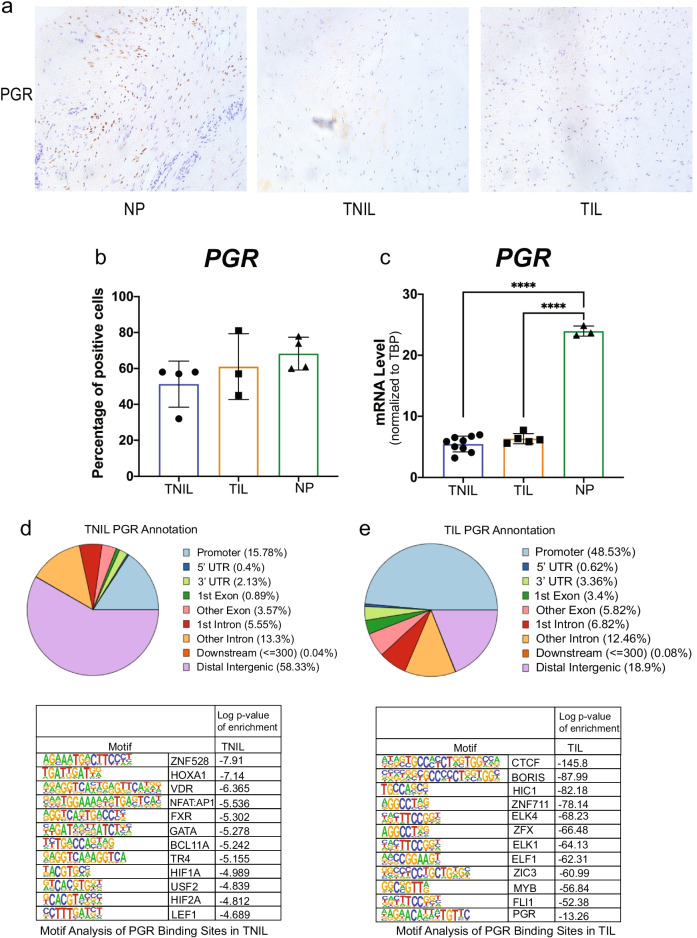

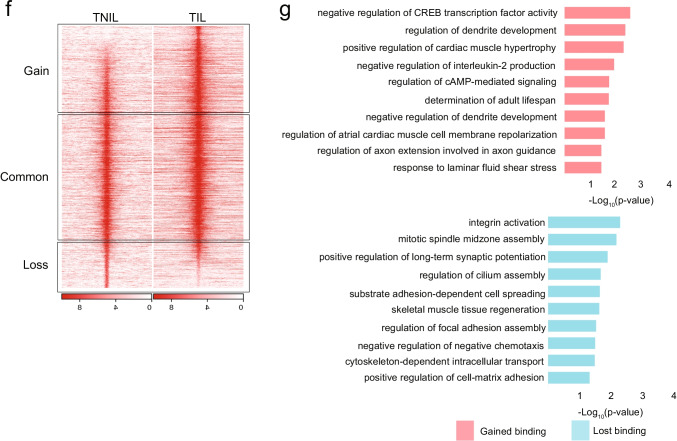
Differential genome-wide PGR binding between term in labor and not in labor myometrium. **a** IHC of myometrial samples from NP, TNIL, and TIL groups for PGR protein. **b** mRNA expression in NP (*n* = 3), TNIL (*n* = 9), and TIL (*n* = 5) human myometrium samples. **c** annotation and motif analysis of TNIL PGR binding sites found from all three ChIP-seq samples. **d** Annotation and motif analysis of TIL PGR binding sites found from all three ChIP-seq samples. **e** Heatmap of TNIL (left panel) and TIL (right panel) binding sites showing gained, common, or lost binding sites in TIL. **f** Gene ontology of genes containing a gained (pink) or lost (blue) PGR binding site in labor

To better understand the molecular mechanisms of the critical roles of the progesterone/PGR pathway in pregnancy and labor, we performed PGR ChIP-seq to profile genome-wide PGR binding in TIL and TNIL myometrial tissues. Prior to that, PGR protein levels were compared between non-pregnant (NP), TIL, and TNIL myometrium using immunohistochemistry (IHC) (Fig. 2a). Immunostaining of myometrium tissues showed lower cell density in TIL and TNIL myometrium versus NP myometrium, probably due to myometrial cell hypertrophy and increased cell volume during pregnancy. Although the staining intensity for PGR protein seemed substantially higher in NP myometrial tissue, the ratio of PGR positive cells to total cells per high-power field was not different between NP, TIL, and TNIL myometrial tissues (Fig. 2b). On the other hand, the tissue mRNA levels of PGR measured by real-time quantitative PCR (RT-qPCR) were significantly lower in TIL or TNIL compared with NP myometrium (Fig. 2c). Taken together, these data suggest that mRNA and protein levels of PGR in myometrial cells were downregulated in pregnant myometrial tissue, which may be associated with the distinct functions of PGR at different reproductive stages through altering its interaction with chromatin.

To assess the genome-wide role of myometrial PGR, myometrial tissues from six individual patients (3 TIL and 3 TNIL) were subjected to PGR ChIP-seq. Consensus PGR peaks in each group were identified and annotated using DiffBind and ChIPseeker, respectively. TNIL samples shared 21,951 peaks and 34,938 peaks were shared in TIL samples. Peak annotation revealed different genomic distributions of PGR binding sites between TIL and TNIL myometrium. PGR preferentially occupied distal intergenic/enhancer regions compared to other genomic regions in TNIL myometrium but not in TIL myometrium (58.33% versus 18.9%). In contrast, PGR bound to promoters preferentially vs. distal intergenic/enhancer regions in TIL myometrium (48.53% versus 15.78%) (Fig. 2d, e), suggesting that PGR binding follows distinct patterns dependent on labor status. In addition, motif analysis uncovered distinct transcription factor binding motifs between these two tissues (Fig. 2d, e). Only 12 motifs were significantly enriched in the TNIL PGR binding sites, whereas 228 motifs were enriched in the TIL PGR binding sites. A classical PGR response element (PRE) was found only among the TIL peaks, where top enriched motifs included CCCTC binding factor (CTCF) and CCCTC binding factor like (BORIS), which play critical roles in chromatin remodeling and epigenetic regulation (Fig. 2e) [14, 23, 24, 42, 48, 56]. Hypermethylated in cancer protein (HIC1) is also involved in chromosomal remodeling and can act as a transcriptional regulator [6, 53]. Binding sequences for several ETS family members (ELF1, ELK1, ELK4, FLI1) were also enriched as the top motifs around PGR binding sites in TIL myometrium; these factors play major roles in various biological processes and are modulated by calcium signaling (Fig. 2e) [9, 11, 27, 37]. These findings suggest that PGR may play distinct roles in TIL and TNIL through interaction with distinct transcriptional factors. Myometrial PGR may be associated more loosely with the intergenic regions in TNIL. In labor, however, PGR may bind to promoter regions and classical progesterone response elements more avidly and participate in chromosomal remodeling and epigenetic regulation.

To more robustly assess the labor status as a determinant for differential genome-wide PGR binding, we used a direct statistical comparison method named DiffBind. DiffBind analysis uncovered over 1700 differential PGR-bound sites between TIL and TNIL, with 1361 sites gained and 428 lost in labor (Fig. 2f). Functional enrichment analysis found pathways involved in cAMP-mediated signaling enriched in labor (Fig. 2g). Genes in this pathway included phosphodiesterase 4A (PDE4A) and phosphodiesterase 10A (PDE10A), a class of enzymes involved in the regulation of intracellular cAMP and cGMP levels necessary for smooth muscle contractility. Selective inhibition of PDE4 has been implemented in smooth muscle relaxation [7, 31, 36, 55]. Cell membrane repolarization, negative regulation of CREB activity, and negative regulation of interleukin-2 production were also uniquely enriched for genes found to have enhanced PGR binding in TIL myometrial tissue (gained binding in labor, Fig. 2g). On the other hand, focal adhesion, integrin activation, cell matrix adhesion, and cytoskeleton transport were enriched for genes that lost PGR binding in labor, suggesting the importance of PGR in maintaining extracellular matrix structure in the TNIL group (lost binding in labor, Fig. 2g).

### Histone Modification Binding Maps in Term Pregnant Myometrium

**Fig. 3:**
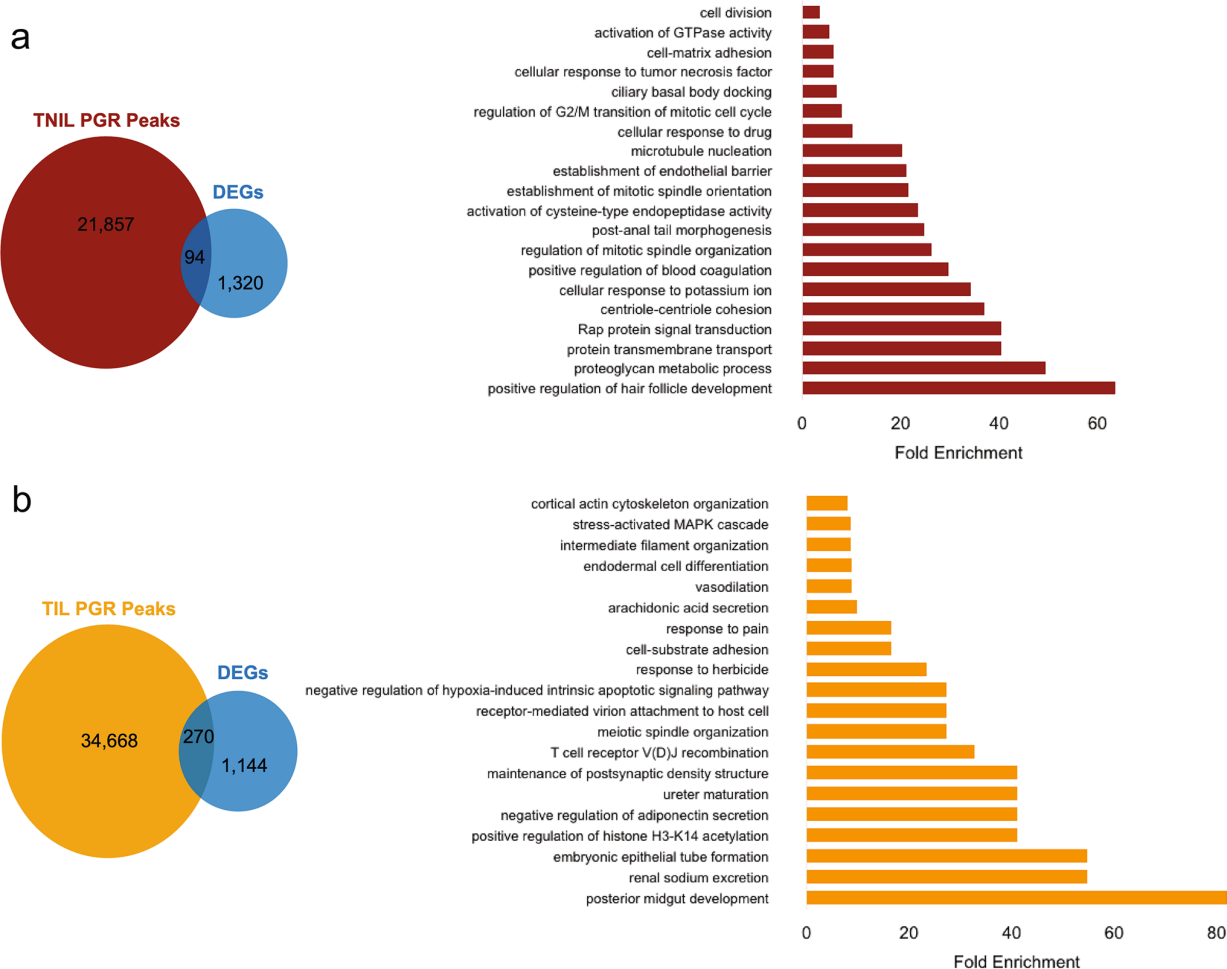
Integration of transcriptome and PGR cistrome uncovers novel target genes in pregnant myometrium. **a** Venn diagram depicting the integration of TNIL PGR binding sites and DEGs (left) and gene ontology. **b** Venn diagram depicting the integration of TIL PGR binding sites and DEGs (left) and gene ontology

**Fig. 4:**
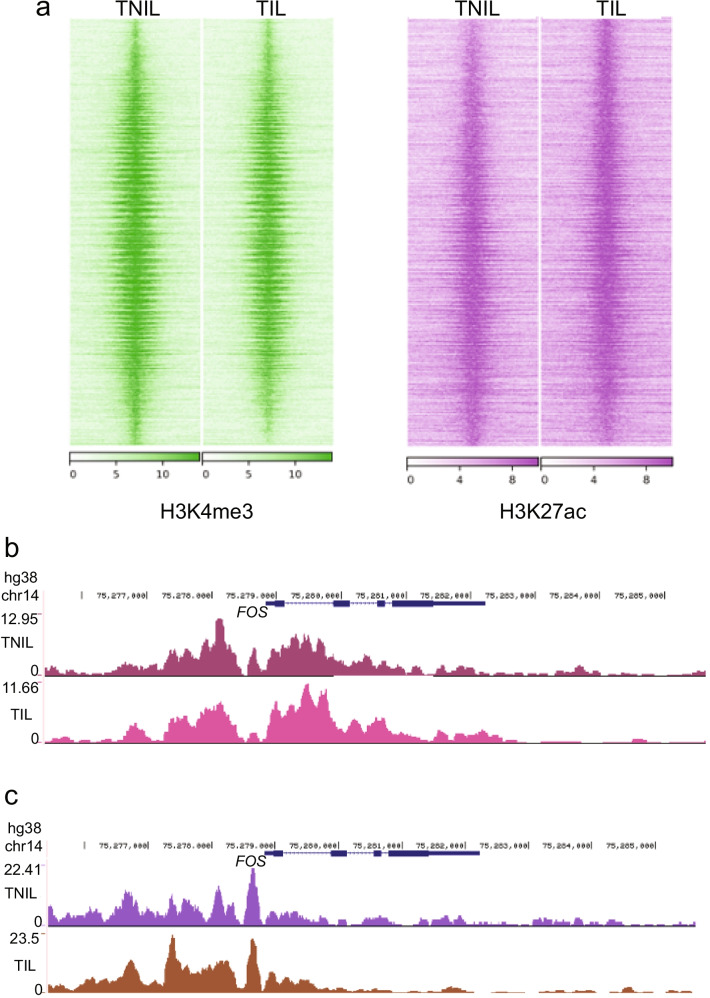

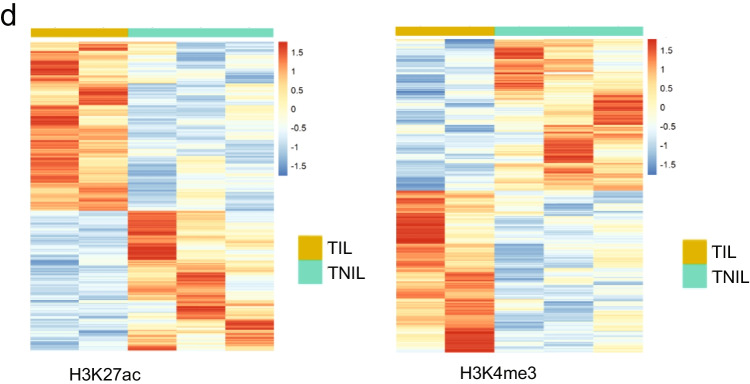
Histone mark binding profile of term laboring and non-laboring myometrium. **a** Heatmap of genome-wide H3K27ac and H3K4me3 binding in TNIL and TIL myometrium. **b** H3K27ac ChIP-seq consensus reads at labor-associated gene *Fos* in TIL (brown) and TNIL (purple). **c** H3K4me3 ChIP-seq consensus reads at labor-associated gene *Fos* in TIL (pink) and TNIL (magenta). **d** Heatmap of differential H3K27ac (left) and H3K4me3 (right) bound regions in individual TIL and TNIL samples

The histone modifications H3K4me3 and H3K27ac are associated with transcriptionally active chromatin (Fig. 3). We performed H3K4me3 and H3K27ac ChIP-seq in TIL (*n* = 2) and TNIL (*n* = 3) myometrial tissues, separate from the samples used for PGR ChIP. We found 15,012 and 14,640 consensus H3K4me3 peaks in TNIL and TIL, respectively. For H3K27ac, 21,856 and 35,441 consensus peaks were found in the TNIL and TIL groups, respectively. Despite differences in the quantity of peaks, genome-wide enrichment of the consensus histone mark peaks shows no major difference between labor statuses (Fig. 4a). A previous study in mice showed that active histone marks are already bound to promoter and enhancer regions of labor-associated genes [45]. Thus, we explored the consensus histone mark signatures at the promoters and distal regions of labor-associated genes: *FOS*, *GJA1*, *OXTR*, and *ZEB1*. Consensus peaks for H3K27ac were found in promoter or enhancer regions of these labor-associated genes (Fig. 4b; Supplemental Fig. 1). Furthermore, H3K4me3 was also found in these labor-associated genes within promoter regions (Fig. 4c, Supplemental Fig. 1). This data supports previous findings from mouse non-laboring uteri that labor-associated genes are activated before labor onset.

Because H3K27ac indicates active transcription at promoters and enhancers, enrichment of transcription factor binding motifs in these regions was performed in TIL and TNIL consensus peaks using HOMER. Promoter regions were further examined for H3K4me3 in TIL and TNIL myometrium. HOMER identified 168, 161, 240, and 241 enriched motifs in H3K27ac distal regions and H3K4me3 promoter regions in TIL and TNIL, respectively (Table 2 and Table 3). Among these motifs, 22 and 15 were uniquely enriched in the H3K27ac distal regions of TIL and TNIL (Table 2) and 49 and 50 were uniquely enriched in the promoter regions of H3K4me3 binding in TIL and TNIL, respectively (Table 3). Most of the transcription factor motifs found for both H3K27ac distal regions and H3K4me3 promoter regions encoded ETS proteins, basic leucine zipper domain (bZIP) proteins, and zinc fingers. Their functions in pregnant myometrium have yet to be elucidated. A portion of transcription factor motifs from TIL PGR peaks (Fig. 2e) overlapped with the H3K27ac and H3K4me3 binding motifs in TIL. Furthermore, the PRE was found in H3K27ac distal binding motifs in both TIL and TNIL myometrium. Therefore, we examined the overlap of PGR binding in pregnant myometrium with H3K27ac distal regions and H3K4me3 promoter regions. Of the 34,938 PGR peaks in labor, only 1.9% of these peaks overlapped with an H3K27ac bound enhancer region (Supplemental Table 4). However, 35.2% of PGR labor peaks overlap with H3K4me3 bound promoter regions (Supplemental Table 5). Taken together, this data suggests a transcriptional activation role of PGR in laboring myometrium that may control the distinct transcriptomes seen by labor status.

**Table 3:** Enriched motifs in TIL and TNIL myometrium H3K4me3 peaks from promoter regions

Group	Histone Mark	Number of Enriched Motifs	List of Uniquely Enriched Motifs
TIL	H3K4me3	240	AMYB, Bapx1, 9cl6, BMYB, CRX, CTCF-SatelliteElement„ EHF, Eames, Fox:Ebox, Foxo1, Hand2, HNF4a, Hoxa11, Hoxa13, Hoxa9, Hoxd11, HRE, Isll1, MafB, Meis1, MYB,Nkx3.1, Nkx6.1, Pitx1, RUNx-AML,, RUNX, RUNX1, RUNX2, SCRT1, Sox10, Sox15, Sox17, Sox2, Sox3, Sox4, Sox6, Sox9, STAT4, Tbet, Tbr1, Tbx21, Tbx6, TEAD1, TEAD3, TEAD4, Tgif2, Lisif 189, ZNF41
TNIL	H3K4me3	241	ArntAhr, bHLHE40, bHLHE41, BORIS, c-Myc, CRE, CTCF(V), DUX, E-box, E2F, E2F1, E2F3, E2F4, E2F6, E2F7, Egrl, Egr2, ELF1, Elk1, ETS, ETV1, ETV4, Fill, HIF-la, HIF-1b, HIF2a, HINFP, KLF10, KLF14, KLF3, Klf4, KLF5, KLF6, Klf9, LRF, Maz, NRF, NRF1„Sp1, Sp2, SO, Tcfcp211, Usf2, WT1, ZBTB12, ZBTB33, Zfp281, ZKSCAN1, ZNF264, ZNF519

**Table 4:** Summary of genes from integration of differential gene expression, PGR occupancy, and H3K27ac occupancy between TIL and TNIL

Differential H3K27ac	Differential PGR Differential Expression		Gene Symbol
Up	Up	Up	COLEC12, ATP11A, NID2, CHST15, KIF5C
Up	Down	Up	TMED10
Down	Up	Down	MPRIP, NCS1, TNS1, ALDH4A1
Down	Down	Down	SVIL, RERG, MAP4, EVA1C, GRIN2A

Up, upregulated or enriched in T1L

Down, downregulated or not enriched in T1L

**Table 5:** Summary of genes from integration of differential gene expression, PGR occupancy, and H3K4me3 occupancy between TIL and TNIL

Differential H3K4me3	Differential PGR	Differential Expression	Gene Symbol
Up	Up	Up	KIF5C
Up	Down	Up	None
Down	Up	Down	TNS1, CBX7, AHDC1
Down	Down	Down	None

Up, upregulated or enriched in TIL

Down, down regulated or not enriched in TIL

Using DiffBind, a portion of consensus peaks were found as differentially bound by the active histone marks in myometrium. We identified 1138 and 2082 genes associated with differential H3K4me3 and H3K27ac enrichment, respectively, between these two tissues (Fig. 4d). Gene ontology revealed pathways related to relaxation of vascular smooth muscle, negative regulation of cAMP-dependent protein kinase activity, and negative regulation of focal adhesion assembly to be enriched among genes with differential binding of H3K27ac (Supplemental Data 2). Moreover, inflammatory response, interleukin-2 mediated signaling, regulation of cardiac muscle contraction by calcium ion signaling, and cell communication by electrical coupling were among some of the pathways enriched from genes found with differential H3K4me3 binding (Supplemental Data 2). This suggests that the specificity of histone binding in pregnant myometrium may have specific functions related to regulation of pathways involved in immune response and smooth muscle contraction [46, 60].

### Integration of Transcriptome and PGR Cistrome Uncovers Novel Target Genes in Pregnant Myometrium

To understand the role of PGR in the regulation of transcripts associated with labor status, we integrated the genes differentially expressed between TIL and TNIL with statistical significance (Fig. 1c) with the PGR binding sites lost in TIL myometrium (Fig. 2f) using CistromeGO [26]. CistromeGo integrates ChIP-seq peaks and differential gene expression to predict the regulatory potential of the transcription factor from the contributing peaks surrounding genes. This analysis uncovered 94 DEGs enriched in biological pathways associated with GTPase activity, cell matrix adhesion, Rap protein signaling, and potassium ion cellular response (Fig. 3a and Supplemental Data 1). In contrast, integration of DEGs with gained PGR binding sites (Figs. 1c and 2f) in TIL yielded 270 genes potentially regulated by enriched PGR binding in labor (Fig. 3b and Supplemental Data 1). These genes were involved in MAPK cascade, histone acetylation, response to pain, negative regulation of hypoxia-induced apoptotic signaling, and cortical actin cytoskeleton organization (Fig. 3b).

To pinpoint functional PGR target genes, we performed a 3-way integration analysis. The DEGs with differential PGR binding (Supplemental Data 1) were integrated with the differential histone modification sites, which revealed 17 candidate genes (Tables 4 and 5). We focused on three genes involved in calcium signaling and actin/myosin pathways because these pathways have importance in smooth muscle contraction. The gene that encodes ATPase phospholipid transporting 11A (ATP11A) was found to be highly expressed in TIL with enriched PGR binding and H3K27ac modification in TIL, suggesting that PGR binding may stimulate its expression in labor [51]. In contrast, CBX7 and TNS1 were downregulated with enhanced PGR binding but decreased active histone (H3K4me3 and H3K27ac) modification binding sites in TIL, suggesting that PGR binding may inhibit their expression [3, 62]. We also noted that KIF5C, a member of the kinesin superfamily of molecular motors and a regulator of local translation, showed increased mRNA levels, PGR binding, and promoter/enhancer histone modifications (Tables 4 and 5) [49]. Although the physiologic roles of KIF5C in uterine smooth muscle are not known, its expression was reported to be upregulated in uterine leiomyomas [61].

### Regulation of PGR Labor-Associated Genes in Human Myometrial Cells

**Fig. 5:**
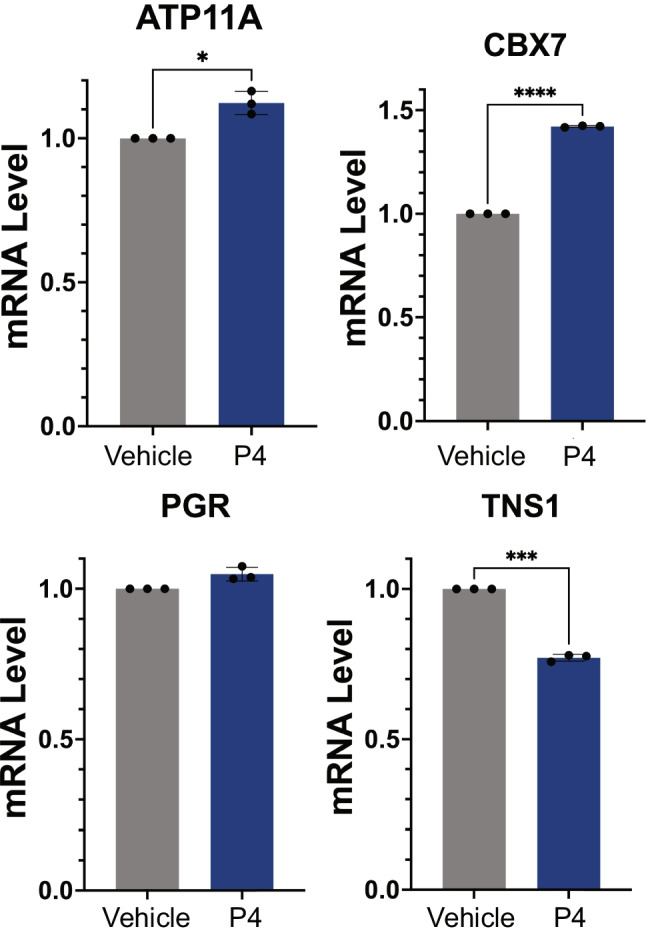
Regulation of PGR labor-associated genes in human myometrial cells. Candidate genes are regulated by P4 in pregnant myometrial cell line. mRNA expression of ATP11A, CBX7, PGR, and TNS1 between vehicle (gray) and 10^−^.^5^ M P4 (blue). Two-tailed Welch’s *t* test was performed for significance. (*P* < 0.05 denoted as *, *P* < 0.001 denoted as ***, and *P* < 0.0001 denoted as ****)

We assessed the regulation of expression of three candidate genes by progesterone using immortalized pregnant human myometrial cells (PHM1-41). The cells were treated with progesterone (P4, 10^−5^ M) for 24 h (*n* = 3). P4 treatment significantly upregulated the mRNA levels of ATP11A and CBX7 and downregulated that of TNS1 expression without any significant effects on PGR mRNA expression (Fig. 5). The results suggest that ATP11A, CBX7, and TNS1 are P4/PGR target genes. The validation studies may best be performed using in vivo experimental systems, which remains outside the scope of this study and should be considered in future directions.

## Discussion

The present study demonstrates the transcriptome and PGR cistrome of TIL and TNIL myometrium highlighting the differences between the two phenotypes. Distinct clustering between the two groups was shown by PCA, and over 1400 significant (FDR < 0.05) DEGs were found, contributing to the transcriptomic differences observed. Parturition-related genes such as OXTR, GJA1, and ZEB1 were not found to be significantly different between labor statuses (Supplemental Table 3). Among statistically significant DEGs (Fig. 1c), calgranulin A (S100A8), calgranulin B (S100A9), IGF-binding protein (IGFBP3), Alpha-actin (ACTA1), GATA Binding Protein 3 (GATA3), and Fms-Like Tyrosine Kinase 1 (FLT1) were present, consistent with another study that determined differentially expressed genes in the myometrium of term women in labor compared to term women not in labor via microarray [33]. In addition, a meta-analysis examining transcriptional differences between term pregnant laboring and non-laboring myometrium found 126 high confidence differentially expressed genes from three datasets [47]. Our differential expression analysis contained 46 of the 126 (Supplemental Table 6). Remarkably, the direction of differential expression was consistent among these genes found in the present study and others [8, 33, 47]. Of those 46 genes, all but one were upregulated. Metallothionein 2A (MT2A), Purine Nucleoside Phosphorylase (PNP), Nicotinamide Phosphoribosyltransferase (NAMPT), Interleukin 1- Beta (IL1B), Triggering Receptor Expressed on Myeloid Cells 1 (TREM1), Serpin Family A Member 1 (SERPINA1), Metallothionein 1X (MT1X), Superoxide Dismutase 2 (SOD2), Solute Carrier Family 7 Member 5 (SLC7A5), Chromosome 15 Open Reading Frame 48 (C15orf48), RAS Like Estrogen Regulated Growth Inhibitor (RERG), Rho Guanine Nucleotide Exchange Factor 37 (ARHGEF37), Transcription Elongation Factor A Like 4 (TCEAL4), Calcium/Calmodulin Dependent Protein Kinase II Gamma (CAMK2G), Glycogenin 2 (GYG2), and Secernin 1 (SCRN1) were among upregulated genes. Protein phosphatase 1 regulatory subunit 3C (PPP1R3C) was the only high confidence gene downregulated between laboring and non-laboring myometrium in our dataset and others.

Gene ontology uncovered that pathways involved in muscle contraction and filament movement processes were enriched in genes highly expressed in TNIL myometrium (Fig. 1d). Many genes encoding for actins, myosin light and heavy chains, tropomyosins, calcium voltage-gated channel subunits, and integrins were enriched within the muscle contraction and actin filament pathways. Calcium supports smooth muscle contraction via binding to calmodulin leading to phosphorylation of the myosin light chain kinase, allowing myosin to interact with actin. Activation of integrins can regulate calcium channels triggering smooth muscle cell contraction and vasoconstriction in isolated arterioles [52, 60]. Because contractions are physically present at the time of collection for the TIL group, the gene expression changes required to alter protein action may have already occurred. Thus, the transcriptomic snapshot taken at tissue collection misses the timepoint in which mRNA levels affect contraction initiation in the TIL group. Inflammatory and immune responses were among the enriched pathways in genes highly expressed in TIL, consistent with previous studies using RNA-seq and microarray [33, 57].

To our knowledge, this is the first comparative analysis of the PGR cistrome in term laboring and non-laboring pregnant human myometrium. PGR cistromic data show differential PGR binding profiles between laboring and non-laboring myometrium (Fig. 2f). Binding in TNIL was substantially different in that it was primarily in the intergenic regions and specific for only 12 motifs, with hypoxia associated motifs overrepresented (HIF1a and HIF2a). In contrast, PGR binding in TIL was more broad, binding to 228 motifs including a classical PRE, and concentrated in the promoter regions. However, the presence of the classical PRE present in the TIL binding sites and not in the TNIL binding sites is inconsistent with PGR occupancy in term pregnant non-laboring myometrium from a recent study [59]. Additionally, intronic regions and enhancers were highly occupied by PGR in term non-laboring pregnant myometrium from the previous study, whereas in our study, mainly distal intergenic/enhancer regions were enriched in term non-laboring pregnant myometrium (58.33%).

Not only is there a difference in the genomic regions in which PGR is binding, but there are also differences in the sequences in which PGR binds in term non-laboring pregnant myometrium (Fig. 2d and e). Our TNIL myometrium lacked numerous transcription factor motifs found in the previous study. Despite these differences, our study found interesting motifs associated with PGR binding in TIL. A portion of these motifs, such as MyoD, STAT, YY1, and SMAD2, overlapped with previously reported motifs in term non-laboring pregnant myometrium [59]. However, CTCF and BORIS motifs were the top enriched motifs found in TIL PGR binding sites. CTCF is involved in forming long-range chromatin loops and acts as an insulator of transcriptional activity [56]. The ETS family of transcription factors (ELK4, ELK1, ELF1, FLI1) were also among the top enriched motifs and play critical roles in various biological processes, including tissue remodeling [11, 21, 37, 44]. Not only were more motifs found in TIL PGR binding sites, but increased enrichment of PGR at TIL motifs compared to TNIL motifs was also observed. Differences between our study and the previous study can be attributed to technical differences in center/hospital tissue collection, sequencing machinery, or data processing [59]. The previous study had two samples, performed 50 nt single-ended sequencing using the Illumina HiSeq 2000 system, reads were aligned using human reference genome hg19. Compared to our three samples, 75 nt single-ended sequencing using the Illumina NextSeq 500 system, and hg38 as the reference genome these technical differences can cause inconsistencies. Furthermore, the differences between studies stress the importance of consistent tissue collection protocols, patient inclusion/exclusion criteria, next-generation sequencing technology, and data processing.

Differential binding analysis for PGR uncovered that pathways involved in cAMP signaling and cardiac muscle cell membrane repolarization were uniquely enriched in PGR binding regions found gained in labor (Fig. 2g). Whereas integrin activation, adhesion-related pathways, and cytoskeleton transport were uniquely enriched for genes that lost PGR binding in labor. These differences suggest that PGR candidate genes in these unique pathways may be important for labor status. It has been reported that PGR isoforms and PGR-A:PGR-B ratio are crucial for the switch from a quiescent phenotype to a contractile phenotype [32, 34, 35, 38, 54]. However, given that the PGR ChIP-seq antibody detects all isoforms of PGR, there is no indication of whether isoform differences contribute to the differential binding seen, limiting our study. Thus, further investigation on the role of the isoforms in human tissue is needed. Overall, the PGR cistromic data sheds light on the targeted pathways and genes in pregnant myometrium that may contribute to the initiation of labor.

Integrative analysis using CistromeGO and differential histone ChIP-seq revealed ATP11A, CBX7, and TNS1 as possible P4 responsive quiescent/contraction associated genes. CBX7 is the gene that encodes the Chromobox 7 protein, a component of a polycomb group, critical for transcriptional repression of many genes, such as the HOX gene family [62]. Interestingly, the expression of CBX7 is downregulated in labor, and inversely upregulated in the non-laboring group, and we found that HOXA1 motifs were enriched in the PGR binding sites from TNIL. ATP11A is an ATPase that catalyzes the hydrolysis of ATP coupled to transporting phosphatidylserines. This phospholipid transporter protein with ATPase activity stimulates calcium influx and Rho GTPase signaling, leading to the assembly of myosin fibers and myotube formation in myoblasts [51]. The upregulation of ATP11A in labor may contribute to the increased assembly of myosin fibers required for myosin and actin cross bridges. Finally, TNS1 has been shown to be involved in actin binding and is essential for myofibroblast differentiation [3]. A TNS1 knockout mouse model was shown to be fertile, yet homozygous female knockout mice produce fewer live progeny than wildtype [29]. This phenotype suggests a role for TNS1 in mammalian reproductive function. Validation of candidate gene hormone responsiveness in vitro using the pregnant myometrial cell line (PHM1-41) yielded conflicting results from our in vitro analysis. Integration data from tissue revealed PGR as a repressor of CBX7 but an activator in vitro. Although tissue culture is a great model for the in vivo environment, previous studies demonstrated that progesterone/PGR signaling needs the tissue intact to fulfill its appropriate genomic function [22, 50]. Thus, the in vitro model cannot fully recapitulate the in vivo condition. Additionally, this in vitro model does not assess functional PGR isoforms. The regulation of candidate genes by PGR isoform should be addressed in future in vitro studies using models that can manipulate isoform levels. For future mechanistic studies, we will collect fresh myometrial tissue from TIL and TNIL patients, treat myometrial tissue explants with progesterone, and determine whether and how fresh TIL and TNIL myometrial tissues respond differentially to progesterone treatment and assess PGR isoform ratio in the tissues. To further the impact of future studies, the inclusion of preterm laboring myometrium is required and should be used in candidate gene validation and additional mechanistic experiments. Taken together, this work provides a critical genome-wide database for future studies. It sets a foundation for research involving PGR in human labor cascade and possible target genes and pathways for therapeutic intervention in myometrial contraction.

## Materials and Methods

### Subject Criteria

Women undergoing cesarean section (C-section) at Prentice Women’s Hospital of Northwestern Memorial Hospital were consented as per a protocol approved by the Institutional Review Board of Northwestern University. Women were categorized under two groups: term not in labor (TNIL; > 37 weeks displaying no signs of cervical dilation or contraction of the uterus) or term in labor (TIL; > 37 weeks). Both groups excluded women undergoing C-sections for fetal or maternal stress, breech presentation, or any infection. After informed consent was obtained from each patient, samples were de-identified once tissue was collected.

### Tissue Collection

Myometrial tissue (2 × 0.5 × 0.5 cm) was excised from the upper rim of the transverse C-section incision made in the lower uterine segment. The muscle tissue was dissected off the serosa (peritoneum) or endometrium. Then it was immersed in 15 mL of ice-cold PBS in a 50 mL Falcon Tube and washed three times with 15 mL of PBS. After washing tissue, samples were snap-frozen using liquid nitrogen and stored at – 80 °C before proceeding with RNA isolation and chromatin isolation, while a small portion of tissue was fixed for immunohistochemistry (IHC).

### Immunohistochemistry

A small portion of tissue was fixed in Davidson’s Fixative overnight and then immersed in fresh 100% ethanol until IHC. Fixed tissues were stained for progesterone receptor (M3569, Agilent Dako) and IgG as a control.

### RNA Isolation

Frozen tissues were homogenized using a mortar and pestle cooled with liquid nitrogen, and approximately 0.35 g to 0.5 g of tissue was used. RNA isolation was performed according to the mini RNeasy kit (74,104, QIAGEN) instructions. RNA concentration and yield were quantified using Nanodrop.

### RNA Sequencing

DNA library for each RNA sample was constructed using the KAPA RNA Hyper Prep Kit in conjunction with the KAPA Single-Indexed Adapter Kit (KAPA Biosystems). For library amplification, 500 ng of RNA was used with an adapter concentration according to KAPA protocol.

### RNA-seq Data Analysis

RNA sequencing (RNA-seq) reads were aligned using STAR aligner (v2.6.3) with default settings [12] to GR38 human genome assembly. Reads count per gene was performed using htseq [1]. Differential gene expression was detected using the Bioconductor package DESeq2 [30] with the threshold of false discovery rate (FDR) at 0.05 level. Expression values were transformed using DESeq2’s regularized log transformation (rlog) before visualization using principal component analysis (PCA).

### Chromatin Immunoprecipitation Sequencing

Chromatin Immunoprecipitation (ChIP), library preparation, and sequencing (ChIP-seq) were performed by Active Motif Services (Carlsbad, CA). In brief, genomic DNA regions of interest from sample chromatin were isolated using an anti-PGR antibody (sc-7208, Santa Cruz Biotechnology). Illumina sequencing libraries were prepared from the ChIP and Input DNAs and sequenced on Illumina’s NextSeq 500 (75 nt reads, single-end).

### ChIP-seq Data Analysis

The 75-nt single-end sequence reads were mapped to the GR38 human genome assembly using Bowtie2 [25] with the in-house script and alignment information for each read stored in the BAM format. Peak calling in the ChIP-seq data for each sample was performed using HOMER [20]: findPeaks (-factor) for PGR ChIP-Seq, and findPeaks (-histone) for H3K27ac and H3K4me3 ChIP-seq. BED files were constructed from the peaks identified by using HOMER (pos2bed function). The BAM and BED files from replicates were used as input to the Bioconductor package DiffBind [41] to identify merged intervals of peaks and consensus overlapping regions across all replicates in the TIL and TNIL groups. ChIPseeker [63] was used to annotate the consensus regions of the TIL and TNIL groups. Differential bound sites in the consensus regions were detected using DiffBind, which combines the results from DESeq2 and edgeR (*P* < 0.05) based on the read counts. The quality of the differentially bound sites was examined by PCA (not shown). In the PGR ChIP-seq data, differentially bound sites with a loading value magnitude greater than 0.005 in the second principal component were considered noisy sites and removed from the set of differential sites. Fisher’s Exact test was used to assess the significance of enrichment or deviation of ChIP-seq signal in each annotated region across the differentially bound sites from the expected signal distribution across the entire set of consensus sites.

Motif analyses in the consensus regions and in the peak regions of each replicate were performed using HOMER (findMotifsGenome.pl) with the region size equal to the size of the peak (-size given). Fluff [15] was used to generate the profile plots of ChIP-seq peaks using the BED files of the consensus overlapping regions from DiffBind and the individual samples’ BAM files (not shown).

### Integrative Analysis of RNA-seq and ChIP-seq Data

Cistrome-GO [26] was used to integrate RNA-seq with PGR ChIP-seq. The output files from DESeq2 on RNA-seq data and the ChIP-seq consensus regions in BED format using the negative log *P* values for the score column were used as input. Cistrome-GO first computes (1) a gene ranking (*R*_*DE*_) in RNA-seq data determined by differential expression level (LogFoldChange) multiplied by the negative Log of adjusted P-values between TIL and TNIL, and (2) a gene ranking (*R*_*RP*_) in a ChIP-seq data set by the adjusted regulatory potential (RP) score, where RP score is defined by ${s}_{g}=\sum_{i=1}^{k}{2}^{-\frac{{d}_{i}}{{d}_{0}}}$ with ${d}_{i}$ the distance between the $i$th peak’s center in the promoter and the transcription start site of a gene and ${d}_{0}$ a parameter with default value for promoter-type transcription factor. It then determines the aggregated gene rank by the product of the two ranks (*R*_*DE*_ *R_*RP*_). The final list of the differentially expressed genes with at least one differential PGR binding site was determined using the criteria that (1) the expression changes between TIL and TNIL (FDR < 0.05), (2) the occupancy changes of PGR between TIL and TNIL at the consensus overlapping sites (RPscore > 0.1), and (3) occupancy changes of at least one histone mark between TIL and TNIL within the gene in the same direction of differential gene expression (Table 4 and Table 5).

### Cell Culture

PHM1-41 cells were received from American Type Culture Collection (ATCC). Cells were cultured in DMEM (ATCC No. 30–2002) supplemented with 2 mM glutamine, 0.1 mg/mL G-418, and 10% heat-inactivated fetal bovine serum (FBS) in 37 °C incubator with 5% CO_2_. Media was refreshed every 2 to 3 days before passaging for experiments. To assess candidate gene regulation by P4, cells were incubated with 10^−5^ M P4 and vehicle for 24 h before RNA extraction and complementary DNA (cDNA) synthesis for real-time quantitative PCR (RT-qPCR).

### Real-Time Quantitative PCR

About 0.5–1 µg of total RNA was reverse transcribed using qScript cDNA Synthesis Kit (QuantaBio, 95,047–100). All TaqMan assays used for this study were purchased from ThermoScientific (Catalog No. 4331182). Results were analyzed in GraphPad Prism version 9.3.1. For analysis of RT-qPCR data, a two-tailed Welch’s *t* test assuming unequal variance was used.

### Data Deposit

The RNA-seq and ChIP-seq raw data and processed files are deposited in the Gene Expression Omnibus of the National Center for Biotechnology Information at the National Library of Medicine under the SuperSeries GSE202029.

## Supplementary Information

Below is the link to the electronic supplementary material.

Supplementary file1 (XLSX 30 KB)

Supplementary file2 (XLSX 61 KB)

Supplementary file3 (EPS 5974 KB)

Supplementary file4 (EPS 6610 KB)

Supplementary file5 (EPS 6826 KB)

Supplementary file6 (EPS 5855 KB)

Supplementary file7 (EPS 4256 KB)

Supplementary file8 (EPS 3847 KB)

Supplementary file9 (EPS 6820 KB)

## Data Availability

The data that support the findings of this work are available from the corresponding author upon reasonable request.

## Code Availability

The code used to analyze the data that support the findings of this work are available from the corresponding author upon reasonable request.
